# Engineering the residual side chains of HAP phytases to improve their pepsin resistance and catalytic efficiency

**DOI:** 10.1038/srep42133

**Published:** 2017-02-10

**Authors:** Canfang Niu, Peilong Yang, Huiying Luo, Huoqing Huang, Yaru Wang, Bin Yao

**Affiliations:** 1 Key Laboratory for Feed Biotechnology of the Ministry of Agriculture, Feed Research Institute, Chinese Academy of Agricultural Sciences, Beijing 100081, People’s Republic of China

## Abstract

Strong resistance to proteolytic attack is important for feed enzymes. Here, we selected three predicted pepsin cleavage sites, L99, L162, and E230 (numbering from the initiator M of premature proteins), in pepsin-sensitive HAP phytases YkAPPA from *Yersinia kristensenii* and YeAPPA from *Y. enterocolitica*, which corresponded to L99, V162, and D230 in pepsin-resistant YrAPPA from *Y. rohdei*. We constructed mutants with different side chain structures at these sites using site-directed mutagenesis and produced all enzymes in *Escherichia coli* for catalytic and biochemical characterization. The substitutions E230G/A/P/R/S/T/D, L162G/A/V, L99A, L99A/L162G, and L99A/L162G/E230G improved the pepsin resistance. Moreover, E230G/A and L162G/V conferred enhanced pepsin resistance on YkAPPA and YeAPPA, increased their catalytic efficiency 1.3–2.4-fold, improved their stability at 60 °C and pH 1.0–2.0 and alleviated inhibition by metal ions. In addition, E230G increased the ability of YkAPPA and YeAPPA to hydrolyze phytate from corn meal at a high pepsin concentration and low pH, which indicated that optimization of the pepsin cleavage site side chains may enhance the pepsin resistance, improve the stability at acidic pH, and increase the catalytic activity. This study proposes an efficient approach to improve enzyme performance in monogastric animals fed feed with a high phytate content.

Phytate is the most common storage form of phosphorus in plant biomass used for food and feed^1^. Under physiological conditions, the negatively charged phytate usually forms an insoluble complex with important mineral ions and proteins^2^. Phytate phosphorus is not digested by monogastric animals and is usually excreted in the feces because of a lack of endogenous phytase in the gastrointestinal tract^3^. The addition of exogenous phytase can improve the efficiency of nutrient utilization, resulting in economic and environmental benefits^4^,^5^,^6^,^7^.

Phytase is a biocatalyst that is able to degrade phytate. Since the first report of phytase in 1907^8^, numerous phytases have been found in bacteria, fungi, plants, and some animals^9^,^10^,^11^. Phytases are grouped into four major classes, histidine acid phosphatase (HAP), cysteine phosphatase (CP), purple acid phosphatase (PAP), and β-propeller phytase (BP), based on their catalytic characteristics^12^,^13^. Most microbial phytases belong to the HAP family^14^.

The HAP phytases possess a large (α/β) domain and a small α domain with the active site motif RHGXRXP for catalysis and HD for substrate binding/product leaving^15^,^16^. Substrate hydrolysis by enzymes in this family occurs via a characteristic two-step mechanism, including a nucleophilic attack of catalytic H on a scissile phosphomonoester and the hydrolysis of a covalent phosphohistidine intermediate with the release of H^15^,^17^. The optimal activity is usually between pH 1.3–5.5^18^,^19^ and 45–70 °C^19^,^20^,^21^, the substrate specificity is diverse^22^ and HAP family enzymes display different tolerance to acidity, heat, and proteolytic digestion^23^,^24^,^25^,^26^.

Phytase protease resistance can affect the *in vivo* activity and efficacy of the enzyme. Protein engineering is an effective technique to produce protease-resistant enzymes. For example, protein surface loops were subjected to saturation mutagenesis to construct a *Bacillus subtilis* lipase mutant with an increased subtilisin digestion half-life of 17-fold (about 16 h at an equimolar lipase/protease ratio at 40 °C)^27^. The proteinase K cleavage site of bovine pancreatic ribonuclease A was replaced with proline to create a mutant enzyme with a proteolysis rate decreased by two orders of magnitude compared to the wild type^28^. The surface positive charge of *Photinus pyralis* firefly luciferase was reduced to generate a mutant enzyme with an increased trypsin digestion half-life of about 4-fold at 23 °C^29^. Site-directed mutagenesis demonstrated that the *N*-glycosylation conferred pepsin resistance on *Yersinia* phytases and stability at acidic pH^30^. Other engineered phytase mutants also showed increased resistance to proteolysis and improved thermostability^31^,^32^,^33^. The protease resistance of an enzyme may be ascribed to substrate rigidity or the conformation of the protease attacking site^27^,^34^,^35^.

In our previous studies, *Y. kristensenii* YkAPPA and *Y. enterocolitica* YeAPPA were sensitive to pepsin after expression in *E. coli*, but *E. coli*-produced *Y. rohdei* YrAPPA was highly pepsin-resistant^21^,^30^,^36^,^37^. In this study, we employed a structure-based rational design approach to engineer a residual side chain in the surface pepsin cleavage site of *Yersinia* phytases to increase enzyme performance. A smaller or more rigid side at the pepsin cleavage site may improve the fitness of an enzyme in gastric protease by increasing its thermostability and its stability at acidic pH.

## Results

### Selection of the phytase pepsin cleavage site

**Figure 1:**
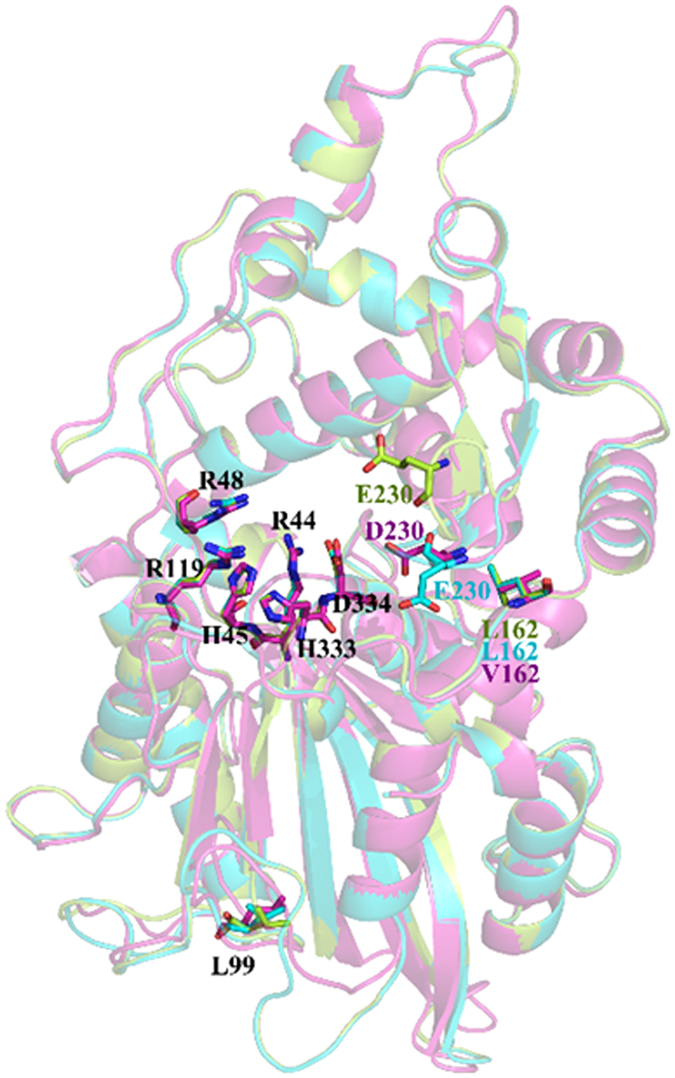
Modeled structures of YeAPPA (green), YkAPPA (blue) and YrAPPA (purple) using *E. coli* phytase (1DKP) as the template with the mutated sites and active sites indicated. Conserved residues in three phytases are shown in black.

Three phytases from *Y. enterocolitica, Y. kristensenii*, and *Y. rohdei*^20^,^36^,^37^ shared more than 83% amino acid sequence identity. Modeled structures of *Yersinia* phytases (Fig. 1) showed three theoretical pepsin cleavage sites: L162 and E230 in YeAPPA and YkAPPA, corresponding to V and D in YrAPPA, respectively, and L99 was conserved in the three phytases. L162 and E230 were substituted by T and S and V and G in pepsin-resistant phytases from *A. ficuum* and *A. niger*, respectively (Supplementary Fig. S1 and Supplementary Table S1). The three sites were situated on different surface loops and assigned different predicted solvent accessibility scores (1, 2, and 4 for L99, L162, and E230, respectively) (Fig. 1 and Supplementary Table S2).

### Production and purification of phytases in *E. coli*

The wild-type and mutant phytases were fused to a C-terminal His6-tag sequence and then expressed under the control of T7 *lac* promoter in *E. coli*. All recombinant enzymes were purified to electrophoretic homogeneity, exhibiting a single band with the theoretical mass (~46 kDa) on SDS-PAGE (Supplementary Figure S2).

### Proteolytic resistance of wild-type and mutant phytases

**Figure 2:**
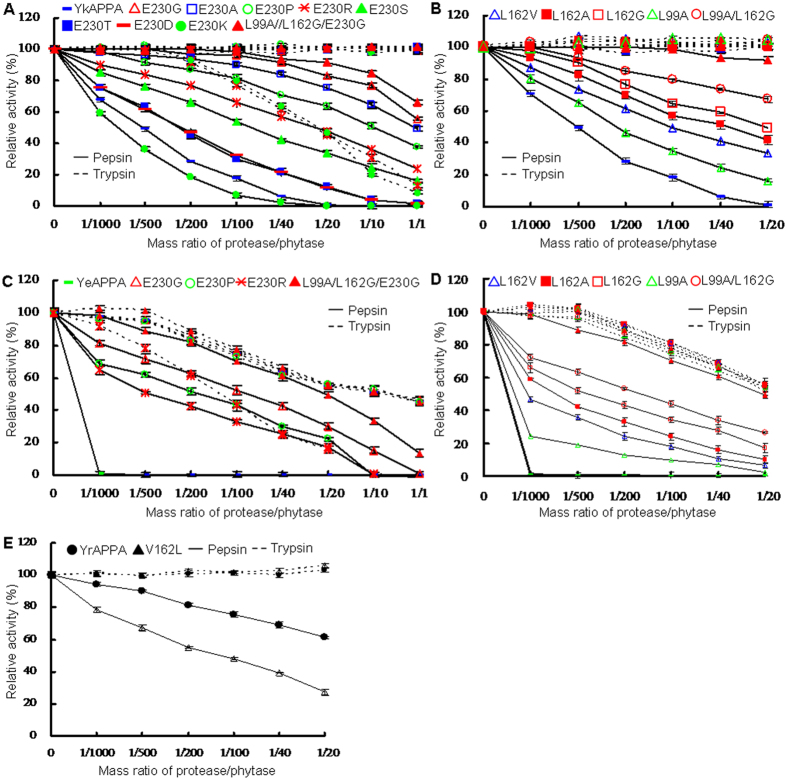
Proteolytic resistance of the wild-type and mutant phytases. (**A**) Protease resistance of wild-type YkAPPA, its single mutants E230G/A/P/R/S/T/D/K, and combination mutant L99A/L162G/E230G. (**B**) Protease resistance of wild-type YkAPPA, its single mutants L162G/A/V and L99A, and combination mutants L99A/L162G and L99A/L162G/E230G. (**C**) Protease resistance of wild-type YeAPPA and its mutants E230G/P/R and L99A/L162G/E230G. (**D**) Protease resistance of wild-type YeAPPA and its mutants L162G/A/V, L99A, L99A/L162G, and L99A/L162G/E230G. (**E**) Protease resistance of YrAPPA and its mutant V162L. Resistance to pepsin at pH 2.0 (full lines) and trypsin at pH 7.0 (dotted lines) was evaluated at various protease/phytase mass ratios and 37 °C for 2 h. The phytase activity toward sodium phytate (1.5 mM) at 37 °C for 30 min was regarded as 100%, and the residual activity is indicated as percentage of activity of untreated enzymes, with means ± SDs of three replicates (n = 3).

The protease resistance of wild-type and mutant phytases were determined over a broad range of protease/phytase mass ratios after incubation at 37 °C for 2 h (Fig. 2). All of the enzymes showed a decreased pepsin resistance with an increase in the pepsin/phytase ratio (Fig. 2A–E). In comparison to YrAPPA, which retained 61.5 ± 0.87% activity even at the highest pepsin/phytase ratio of 1/20 (Fig. 2E, full line), YkAPPA and YeAPPA were much more sensitive to pepsin at all mass ratios tested (Fig. 2A–D, full line). When treated with trypsin at various ratios from 1/1000 to 1/20, YkAPPA (Fig. 2A,B, dotted line) and YrAPPA (Fig. 2E, dotted line) and their mutants retained almost all of their activity, while YeAPPA lost 0–46% activity (Fig. 2C,D, dotted line). These results indicated that YrAPPA, which contained fewer pepsin cleavage sites, is highly resistant to pepsin and trypsin digestion, while YkAPPA and YeAPPA, which have two more pepsin cleavage sites than YrAPPA, are resistant to trypsin at a low mass ratio but highly sensitive to pepsin at any tested mass ratio.

Proteolytic resistance can be improved by disturbing the residual side chain preference of a protease. The three surface pepsin cleavage sites (L99, L162, and E230) in YeAPPA and YkAPPA were respectively substituted with residues of different size, shape, and polarity. Various amino acid substitutions at a single pepsin cleavage site had different effects on the protease resistance of all *Yersinia* phytases tested (Fig. 2A–E). After treatment with pepsin in 0.25 M glycine-HCl (pH 2.0) for 2 h, the residual activities of YkAPPA and its E230 mutants followed the order E230G (83.0 ± 1.1%, P < 3.7E-4) > E230A (75.5 ± 1.9%, P < 3.9E-4) > E230P (63.6 ± 2.1%, P < 4E-4) > E230R (48.5 ± 2.4%, P < 1.8E-4) > E230S (33.7 ± 1.9%, P < 0.0020) > E230T (12.4 ± 1.4%, P < 0.0219) and E230D (11.7 ± 1.5%, P < 0.0273) > YkAPPA (1.1 ± 1.5%) > E230K (0 ± 0.29%, P < 0.3251 versus the wild type) at a pepsin/phytase mass ratio of 1/20 (Fig. 2A). In comparison with pepsin-sensitive YeAPPA, E230G, E230P, and E230R mutants of YeAPPA had increased pepsin resistance (447 ± 7.8-, 315 ± 6.7-, and 265 ± 5.5-fold, respectively, at a ratio of 1/40) (Fig. 2C and Supplementary Table S4). After deleting a single pepsin cleavage site at various positions, the residual activities of pepsin-treated YeAPPA, YkAPPA and the mutants L99A, L162V, L162A, and L162G are the lowest to highest at ratios from 1/1000 to 1/20 (Fig. 2B,D and Supplementary Table S4). In contrast, when a single pepsin cleavage site was introduced into a pepsin-resistant YrAPPA, YrAPPA-V162L only retained 27.3 ± 2.1% of the activity at a ratio of 1/20, much less than the wild type (61.3 ± 0.87% activity, P = 0.0021) (Fig. 2E). When treated with trypsin in 0.25 M Tris-HCl (pH 7.0) for 2 h, E230K and E230R in YkAPPA and E230R in YeAPPA retained less activity than the wild type at ratios ranging from 1/200 to 1/1 (P < 0.0031) and 1/1000 to 1/1 (P < 0.0064), but the activity in the other mutants was similar to the wild type at the various ratios (P > 0.1597) (Fig. 2A–D). The results indicated that residues at positions 99, 162, and 230 in *Yersinia* phytases play a major role in pepsin resistance, especially position 230.

Combined substitution in L99A, L162G and E230G was also conducted to assess the additive effect of the deletion of pepsin cleavage sites on protease resistance. After pepsin treatment for 2 h, the double mutants L99A/L162G retained a higher residual activity (≥67.4 ± 2.0% activity for YkAPPA- L99A/L162G [P < 0.0085] and ≥26.2 ± 0.41% activity for YeAPPA-L99A/L162G [P < 0.0057]), at pepsin/phytase ratios ranging from 1/500 to 1/20 than the single mutants (Fig. 2B,D). In the presence of trypsin, the activity in L99A/L162G was similar to wild type (P > 0.1195) (Fig. 2B,D). These results indicated an additive effect of pepsin cleavage site deletion on pepsin but not trypsin resistance. This conclusion was further verified in the triple mutants L99A/L162G/E230G, which retained greater residual activity than the single and double mutants after 2 h of pepsin treatment (P < 0.0179 versus YkAPPA-E230G at the ratios ranging from 1/20 to 1/1, P < 0.0076 versus YkAPPA-L99A/L162G at the ratios ranging from 1/500 to 1/1, and P < 0.0054 for YeAPPA-E230G and YeAPPA-L99A/L162G at all ratios tested (Fig. 2A–D and Supplementary Table S4).

**Figure 3:**
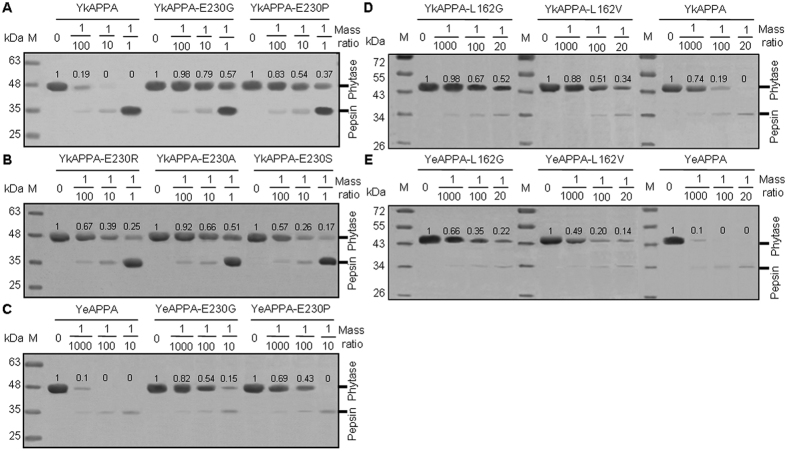
SDS-PAGE analysis of the proteolytic products of wild types and mutants of YkAPPA (**A**,**B**,**D**) and YeAPPA (**C**,**E**) after pepsin treatment at pH 2.0 and 37 °C for 2 h at various pepsin/phytase mass ratios. The phytase band intensity was evaluated by using the ImageJ software. M indicates the standard molecular weight markers. Pepsin has a molecular mass of about 35 kDa.

Further SDS-PAGE analysis confirmed a functional role against pepsin digestion for the residues at positions 99, 162, and 230 (Fig. 3 and Table 1). After incubation with pepsin for 2 h, the single mutants YkAPPA-E230G/A/P/R/S, YeAPPA-E230G/P, YeAPPA-L162G/V and YkAPPA-L162G/V displayed greater tolerance to pepsin digestion, showing slower degradation than the wild type as the mass ratio increased from 1/100 to 1/1, 1/1000 to 1/10, and 1/1000 to 1/20, and the E230G mutants of YkAPPA and YeAPPA had less degraded protein with a higher relative retention value of 0.79 at a ratio of 1/10 and 0.82 at a ratio of 1/1000, respectively, than that for the other single mutants of YkAPPA and YeAPPA, which had a relative residual value of ≤0.69 at the ratios of 1/10 and 1/1000, respectively) (Fig. 3A–E). The single mutants E230G/A/P/R/S and L162G/A/V showed a decreased proteolytic rate of two to three orders of magnitude, and E230D/T and L99A had a reduced proteolytic rate of 8- to 54-fold except for E230K, which showed a slightly increased proteolytic degradation rate of 2.9-fold (Table 1). Although YkAPPA-E230K had decreased proteolytic half-life of 3.5-fold, the other single mutants of YkAPPA and YeAPPA had an increased half-life of 3- to 1924- and 40- to 1700-fold, respectively (P < 0.0024, versus 0.007 ± 2E-4 × 10^−4^ g for YkAPPA and 0.0001 ± 1E-4 × 10^−4^ g for YeAPPA) (Table 1). A combination of L99A, L162G, and/or E230G had an additive effect on proteolytic rate and half-life. Proteolytic rate after pepsin treatment was lower for L99A/L162G than for its single mutants and for L99A/L162G/E230G than for L99A/L162G and E230G. L99A/L162G and L99A/L162G/E230G showed a longer proteolytic half-life than the single and double mutants, respectively (Table 1).

### Effect of pH and temperature on the activity and stability of wild-type and mutant phytase

The pH and temperature resistance of purified mutant phytases were compared with the wild type (Table 2). All of the phytases had similar pH optima (pH 4.0–5.0) with a downward shift up to 0.5 pH units. After 1 h incubation at pH 1.0, the E230G/A/R/K, L162G/V/A, L99A/L162G, and L99A/L162G/E230G variants of YkAPPA retained more than 80% activity while the residual activity of the E230P/S/T/D, L99A variants and the wild-type YkAPPA dropped to approximately 64%. All YeAPPA mutants were more stable than the wild type at pH 1.0–2.0 (P < 0.0216).

**Figure 4:**
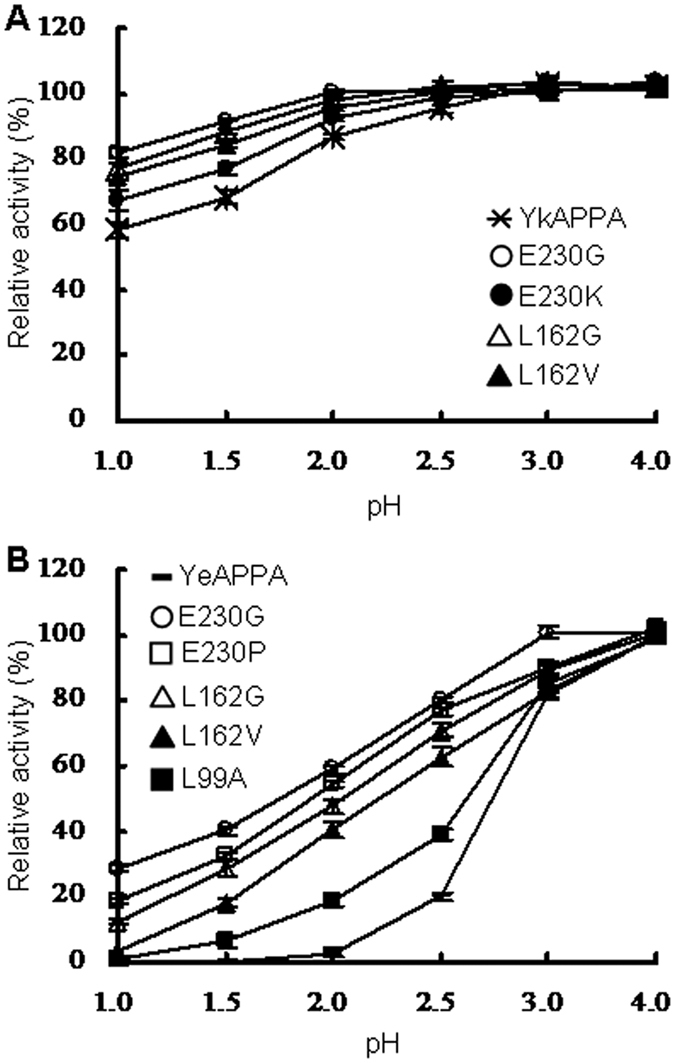
Phytase stability of YkAPPA, YeAPPA and their variants at acidic pH (1.0–4.0) and 37 °C for 2 h. Residual activities of wild types and mutants of YkAPPA (**A**) and YeAPPA (**B**) after acid treatment were calculated as for Fig. 2; Values are indicated as the averages of three independent tests (mean ± SD, n = 3).

The effect of mutations in the pepsin cleavage site on phytase stability at acidic pH was also evaluated at pH values ranging from 1.0–4.0 at 37 °C for a long duration (2 h). As shown in Fig. 4, all phytases showed a pH-dependent decrease in activity. However, the single variants E230G and L162G/V in YkAPPA and YeAPPA, E230K in YkAPPA, and E230P and L99A in YeAPPA lost activity at a much slower rate than the wild type under extremely acidic conditions of pH 1.5–2.5 (P < 0.0084). The results indicated that residue substitution at position 99, 162, and 230 accounts for the appreciable stability of mutant phytases at acidic pH.

The temperature optima of all mutant phytases except for YkAPPA-E230P, YkAPPA-L162V, and YeAPPA-E230P were similar to that of the wild type at 55 °C for YkAPPA and 45 °C for YeAPPA (Table 2). The temperature optimum of YkAPPA-E230P and YkAPPA-L162V was 60 °C and of YeAPPA-E230P was 50 °C, which each was 5 °C higher than the wild type. The thermostability of *Yersinia* phytases varied greatly. After 30 min incubation at 60 °C, the residual activity in YkAPPA and its variants followed the order of E230P (41.7 ± 1.74%) > L99A/L162G/E230G (35.1 ± 1.31%), E230G (34.7 ± 1.23%) > E230S (33.6 ± 1.82%), E230T (33.4 ± 2.42%) > L162V (30.5 ± 1.45%) > E230R (22.6 ± 0.83%) > E230A/D/K, L162A/G, L99A, L99A/L162G, and YkAPPA (15.8 ± 0.48 to 16.8 ± 0.43%) (Table 2). At 60 °C for 30 min, the thermostability of YeAPPA and its variants followed the same order as the wild-type and mutant YkAPPA except for YeAPPA-L99A/L162G/E230G and YeAPPA-E230G, which retained more activities than the other mutants of YeAPPA (Table 2). The half-life in E230G/P/R, L162V, and L99A/L162G/E230G in YkAPPA and YeAPPA and E230S/T in YkAPPA increased up to 12.5-fold at 60 °C (Table 2). Therefore, replacing E230 and L162 in the phytase with G, R, or V not only enhanced the pepsin resistance but also improved their stability at acidic pH and their thermostability.

### Effect of metal ions and chemical reagents on wild-type and mutant phytases

Supplementary Table S5 shows that various metal ions and chemical reagents had different effects on phytase activity. Ca^2+^ enhanced YeAPPA activity by 30 ± 2% but slightly inhibited YkAPPA activity by 6 ± 2%, while Hg^2+^, Fe^3+^, Cu^2+^, Zn^2+^, Pd^2+^, Ag^+^, and SDS strongly inhibited YkAPPA and YeAPPA activity by up to 100 ± 3%. Other chemicals had little or no effect on the activities of both wild-type phytases (P > 0.1284). The L162G and L162V mutants of YkAPPA and YeAPPA showed improved resistance to Hg^2+^, Fe^3+^, Cu^2+^, Zn^2+^, and Ag^+^ (P < 0.0063), and the E230G and E230A mutations in both enzymes alleviated the inhibitory effect of Zn^2+^ (P < 0.0034 versus the wild type). The L99A mutant showed no changes in chemical resistance (P > 0.1946 versus the wild type).

### Kinetic characterization of wild-type and mutant phytase

The kinetic constants of the pepsin cleavage site substitution mutant phytases on phytate sodium hydrolysis were determined and compared with that of the wild type (Table 3). Residue substitution in the pepsin cleavage site had no effect on substrate affinity (P > 0.1674). E230G caused the most significant effect of all of the mutations on the reaction velocity and the turnover rate (up to 2.4-fold). As a result, the catalytic efficiencies of YkAPPA-E230G and YeAPPA-E230G were improved by 2.1- and 2.5-fold, respectively. The L162G mutation in YkAPPA and YeAPPA increased the catalytic efficiency, the reaction velocity and the turnover rate by less than 1.9-fold. The E230A and L162V mutations only enhanced the catalytic performance of YeAPPA, i.e., the reaction velocity, the turnover rate and the catalytic efficiency, but to a much lower level (approximately 1.4-fold). The single mutations L99A, L162A, and E230P/R/S/T/D/K and a combined mutation of L99A, L162G, and E230G had no effects on the substrate affinity, the turnover rate, the turnover number, and the catalytic efficiency of the *Yersinia* phytases (P > 0.1247).

### Enzymatic hydrolysis of corn meal

**Figure 5:**
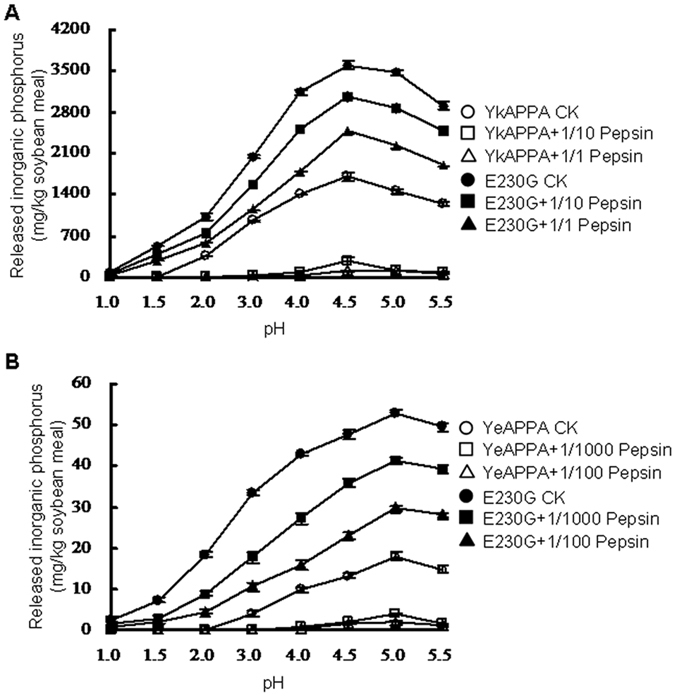
The hydrolysis of corn meal by wild types and E230G mutants of YkAPPA (**A**) and YeAPPA (**B**) after incubation at 37°C for 2 h at pH 1.0 to 5.5 with or without pepsin at various pepsin/phytase mass ratios of 1/1 and 1/10, 1/100 and 1/1000, respectively. Data are indicated as means ± SDs from three replicates (n = 3).

YkAPPA, YeAPPA and their E230G variants varied in the hydrolysis efficacy of a corn meal substrate over a wide range of acidic pH values and at different protease/phytase mass ratios following incubation at 37 °C for 2 h (Fig. 5). Without pepsin addition, the E230G variants of YkAPPA and YeAPPA released more inorganic phosphorus than the wild type at acidic pH, with an increased maximum up to 2.1- fold at pH 4.5 and 2.9- fold at pH 5.0, respectively (Fig. 5A,B). After pepsin treatment in 0.25 M glycine-HCl (pH 1.5–5.5) at various ratios, the hydrolytic efficacy of YeAPPA-E230G and YkAPPA-E230G decreased at a lower rate than that of the wild type and showed an increase from 10.8 to 15.8-fold and from 11.3 to 23.5-fold in the maximal inorganic phosphorus release at pepsin/phytase ratios ranging from 1/1000 to 1/100 and from 1/10 to 1/1, respectively (Fig. 5A,B).

## Discussion

HAP phytases that can improve the production efficiency of inorganic phosphorus in biomass degradation can also maximize livestock profits and offer environmental benefits^38^. Because the functionality of feed enzymes is usually negatively affected by proteases^39^, their proteolytic tolerance in a protease-rich animal digestive tract is critical for their use as a feed additive. Rational protein engineering can improve protein traits through structural analysis, function prediction, and efficient screening^32^,^40^,^41^. This approach has been carried out to enhance the proteolytic tolerance of various natively folded enzymes via the inhibition of protease activity or restricting the enzyme fluctuations at protease-attacking regions^42^,^43^,^44^. Although a few engineered feed enzymes show improved proteolytic resistance^29^,^32^, their resistance with respect to pepsin is still not sufficiently high. Pepsin preferentially cleaves peptide bonds at the carboxylic side of F and L and, to a lesser extent, E linkages, but does not cleave at V, A, or G^45^,^46^. Our previous results have shown that the introduction of *N*-glycosylation sites into *Yersinia* phytase can reduce the accessibility of pepsin to the cleavage site^29^. In this study, we obtained insight into the improvement of pepsin resistance of HAP phytase via another strategy, i.e., substitution of the pepsin cleavage sites L99, L162, and E230 of *Yersinia* phytases with smaller side chains (G, A, D, S, or T at position 230, G, A, and V at position 162, and A at position 99) or more rigid side chains (P or R at position 230).

All pepsin-sensitive YkAPPA and YeAPPA mutants except for YkAPPA-E230K showed obviously enhanced pepsin tolerance, and double mutants of L99A/L162G had greater pepsin resistance than the corresponding single mutants (Figs 2 and 3 and Supplementary Table S4). Similar results were obtained by replacing the pepsin cleavage sites L197 and L396 of *E. coli*-produced *Yersinia* phytases with V, which increased their pepsin resistance digestion^29^. In contrast, the introduction of a pepsin cleavage site at position 162 in YrAPPA lowered its pepsin resistance (Fig. 2E and Supplementary Table S4). Thus, reducing the number of pepsin cleavage sites may improve enzyme pepsin resistance.

**Figure 6:**
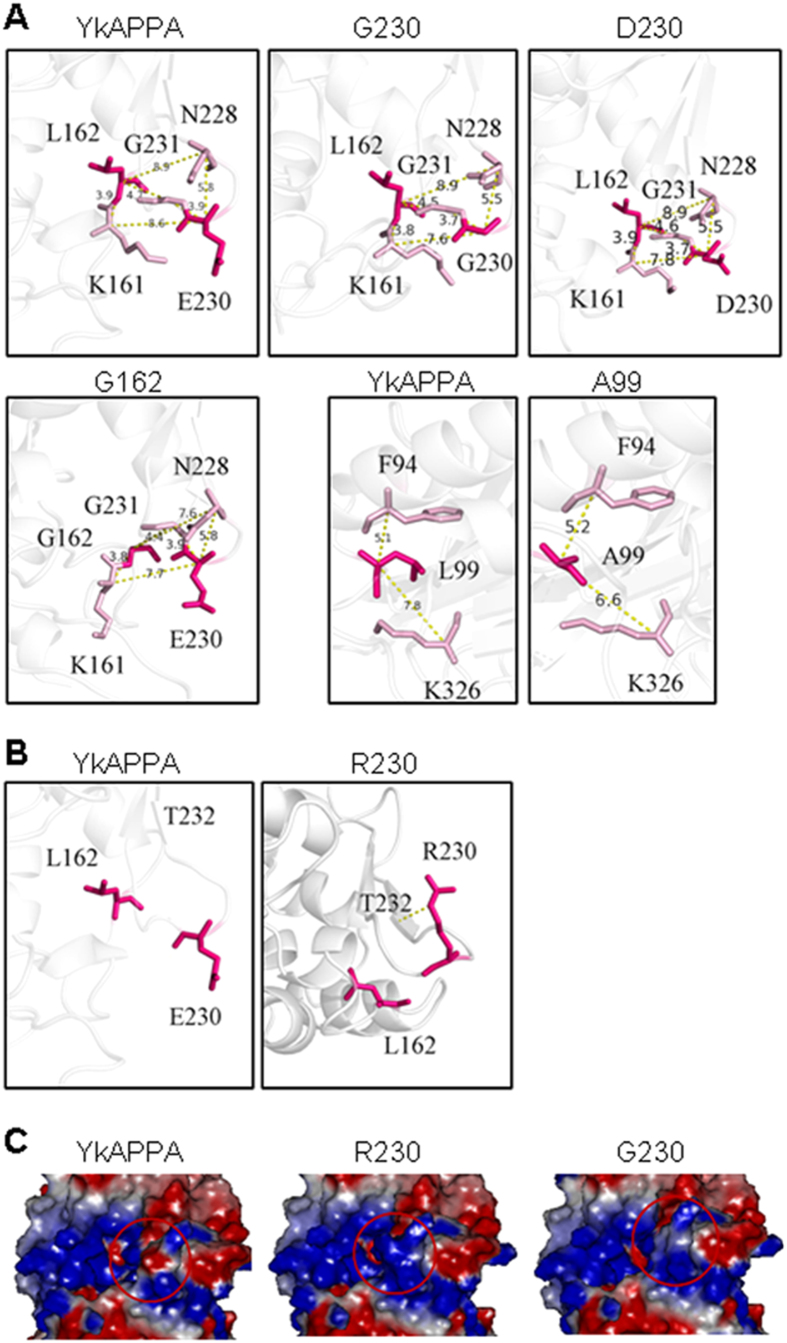
The surface structures of wild-type YkAPPA and its mutants. (**A**) The distances in yellow dash from wild-type E230, L162, and L99 and their mutants G230, D230, G162, and A99 in magenta to the proximate residues K161, N228, G231, and K326 in pink, respectively. (**B**) The effect of E230R mutation on hydrogen bond (yellow dash) and side chain feature. Left panel, wild-type E230; right panel, R230 mutant. (**C**) Electrostatic surface was drawn with Discovery studio 2.5.5 software. The negative charge is red, positive is blue, and neutral is white. The red circle indicates the position 230.

Single substitution at the pepsin cleavage sites of *Yersinia* phytases conferred pepsin resistance, but it varied according to the side structure (Figs 2A–D and 3A–E, and Supplementary Table S4). Modeling of the structures of the wild-type phytase and mutants revealed a shorter distance, 6.6, 7.6, 4.4, 7.6–7.8, and 5.5 Ǻ for A99 with K326G, G162 with N228 and G231, and D/G230 with N228 and K161, respectively, than that in wild types (7.8, 8.9, 4.7, 8.6 and 5.8 Ǻ for E230, L162, and L99 with the proximate residues; Fig. 6A). Thus, the smaller residues G, A, V, S, T, or D at the altered sites in the mutants, compared to wild-type E or L, could reduce some inner space in the phytase structure but open a space between the enzyme and pepsin, thereby decreasing the affinity of pepsin for the enzyme and producing resistance^35^. In contrast, the positively charged larger K residue at position 230 could create a protrusion of the enzyme to narrow the pepsin-binding space and thereby increase the contact with pepsin, improving the cleavage. Although a positively charged R is larger, E230R had an increased pepsin resistance (Figs 2A,C and 3B, and Supplementary Table S4). The side chain of wild-type E230 stretches away from the protein surface (Fig. 6B, left panel), but the guanidinium group enables the R230 side chain to form a new hydrogen bond with T232 and flip onto a β-strand of the mutant protein by approximately 180 °C (Fig. 6B, right panel), which accordingly reduces the contact of phytase with the pepsin, improving pepsin resistance^47^. The pyrrole ring of P230 was presumed to increase its conformational rigidity and sterically protect enzymes against cleavage by pepsin^48^. The improved pepsin resistance is presumably due to the optimization of the side chain structure and the increased conformational rigidity of the pepsin-attacking structural region^42^.

All mutations were also evaluated for their effects on the trypsin resistance of phytases. After trypsin treatment at ratios ranging from 1/1,000 to 1/1, no mutation except for E230K/R changed the trypsin resistance of YkAPPA and YeAPPA. The substitution of K and R for E at position 230 caused decreased trypsin resistance (Fig. 2A–E). Thus, shortening the side chain and reducing the size of the pepsin cleavage site of *Yersinia* phytases may improve their cleavage by pepsin but not by trypsin.

A desirable commercial phytase should be stable at a high pelleting temperature and a low gastric pH and be highly resistant to metal ions. Substitution of the pepsin cleavage site in *Yersinia* phytases improved the thermal and pH stability as well as the protease resistance (Table 2). The combination of two or three pepsin cleavage sites had an additive effect on the improvement of the low pH properties (Table 2). For example, in comparison to the wild-type, YkAPPA, which lost nearly 40% activity at pH 1.0, had a half-life of 12.5 ± 0.52 min at 60 °C and was sensitive to pepsin. The YkAPPA mutants E230G/R had higher stability at acidic pH (92.4 ± 2.5 and 86.8 ± 1.9% of the activity at pH 1.0 for 1 h, respectively), higher thermostability (a half-life of 21.7 ± 0.82 and 14.5 ± 0.37 min at 60 °C, respectively), and increased pepsin resistance (74.0 ± 2.4-fold and 43 ± 1.9-fold, respectively, at a ratio of 1/20) (Fig. 2A and Table 2; see also Table S4). Optimizing the side chain structure of E230G/R (Fig. 6A, middle upper panel; Fig. 6B, right panel), increasing the positive surface charge of E230R (Fig. 6C, middle panel), and decreasing the negative surface charge of E230G (Fig. 6C, right panel) may improve the stability of the enzyme to high temperature and low pH^49^,^50^,^51^. Metal ions can bind to proteins at some atoms, i.e., C and H, inhibiting enzymatic activity^52^,^53^,^54^. The L162A/G mutations alleviated the inhibition of *Yersinia* phytases by Hg^2+^, Fe^3+^, Cu^2+^, Zn^2+^ and Ag^+^ (Supplementary Table S5). The conformational changes induced by mutation were presumed to interfere with binding to metal ions. Thus, optimization of the residual side chains gives *Yersinia* phytases increased pepsin resistance, higher thermostability and stability at acidic pH and insensitivity to some metal ions.

**Figure 7:**
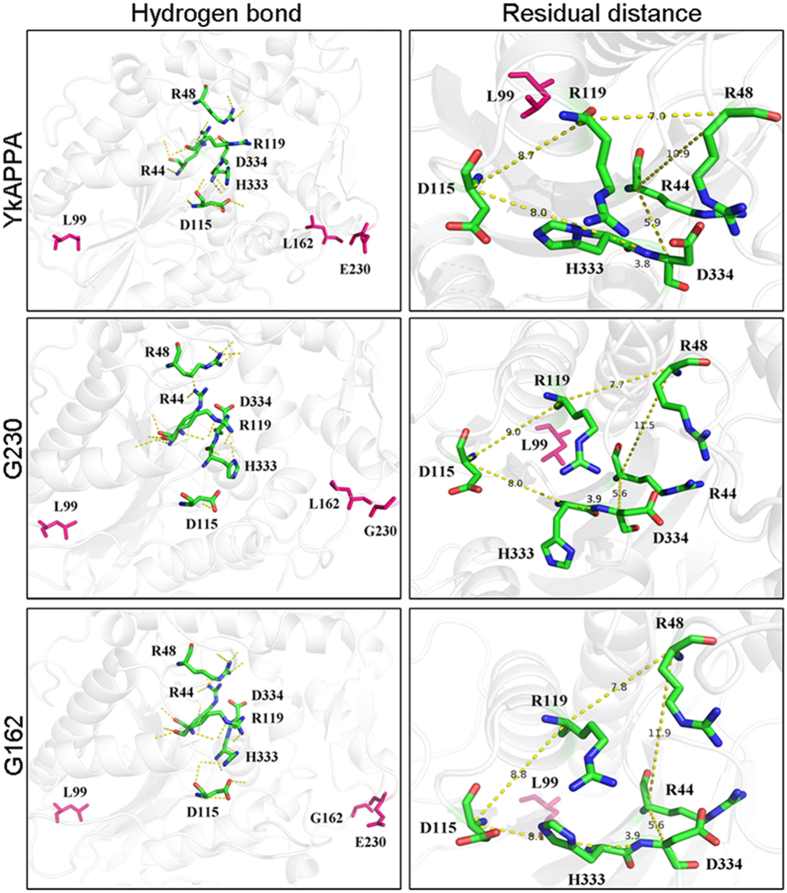
The catalytic center structures of wild-type YkAPPA and its mutants E230G and L162G. The hydrogen bonds around catalytic sites (R44, R48, D115, R119, H333, and D334 in green stick) are shown in yellow dashes (left panels). The distances between six catalytic sites are labeled with different distance values on the yellow dashes (right panels). The key residues are in magenta.

The optimization of the residual side chains also improved the catalytic performance of *Yersinia* phytases (Table 3). Three mutated sites, E230, L99, and L162, occur on the enzyme surface, which is far away from the active sites in *Yersinia* phytases: R44, H45, R48, R119, H333, and D334 (Fig. 1). The structural changes in the catalytic center between the wild-type phytase and mutants are shown in Fig. 7. The catalytic center of E230G and L162G had more hydrogen bonds than the wild type (16 and 19, respectively, versus 13 hydrogen bonds of YkAPPA; Fig. 7, left panels). The distances of R48 with R44 and R119 in E230G (7.7 and 11.5 Ǻ) and L162G (7.8 and 11.9 Ǻ) were greater than in the wild type (7.0 and 10.9 Ǻ for R48 with R44 and R119, respectively), and the angles from D334 to H333 and R44 in both mutants were also increased, giving the catalytic center a larger volume (Fig. 7, right panels). The E230G and L162G mutants may have a larger steric space in the catalytic pocket, which reduces the flexibility of the active residual, increases substrate entry and binding and promotes the catalytic reaction. The improved kinetic properties of YeAPPA by mutations at positions 162 and 230 are probably due to some subtle adjustments caused by the remote residues on the active site after mutation^55^.

The hydrolysis of corn meal phytate by phytase confirmed that the enzyme performance was improved. The stomach is the main functional site of animal feed phytases. The *in vivo* hydrolysis efficiency of phytate catalyzed by phytase commonly occurs at the body temperature of around 37 °C, low pH and high pepsin concentrations in the digestive tract of monogastric animals^56^. The E230G variants of the *Yersinia* phytases had higher hydrolytic efficiency for corn meal phytate into inorganic phosphorus than the wild type under simulated gastrointestinal conditions (Fig. 5). The increased hydrolytic efficacy of the E230G variants was presumably due to their pepsin resistance, stability at acidic pH, and improved catalytic efficiency. Thus, the E230G-containing phytases facilitate the efficient degradation of phytate in plant biomass and have wide biotechnological applications in the animal feed industry.

## Conclusions

Three predicted pepsin cleavage sites, L99, L162, and E230, in *Yersinia* phytases were subjected to protein engineering to improve resistance to proteolysis. The most beneficial E230G/A and L162G/V mutations not only improved pepsin resistance but also caused concomitant improvements in catalytic performance, thermostability, stability at acidic pH, and insensitivity to some metal ions. Mutations confer pepsin resistance by optimizing the side chains of exposed residues. This study proposed a new strategy to improve pepsin resistance and to increase the fitness of phytases for biomass hydrolysis, as well as provided some engineered phytases with excellent properties as candidate feed enzymes.

## Methods

### Strains, vectors, and chemicals

The prokaryotic expression vector pET-22b(+) was purchased from Novagen (Darmstadt, HE, Germany). *E. coli* Trans1-T1 and BL21 (DE3) cells (Tiangen, Beijing, China) were used as the host strain for plasmid amplification and prokaryotic expression, respectively. The restriction endonucleases, *LA Taq* DNA polymerase, and DNA purification kit were obtained from TaKaRa (Otsu, Shiga, Japan). T4 DNA ligase was purchased from New England Biolabs (Beverly, MA, USA). (UK). Phytate (sodium salt) and pepsin (P0685) were ordered from Sigma-Aldrich (St Louis, Mo, USA). All chemicals used in this study were of analytical grade.

### Selection of the mutagenesis residues in phytases

The sequence alignment of the phytases from *Y. kristensenii, Y. enterocolitica, Y. rohdei, Aspergills ficuum*, and *A. japonicus* was analyzed with the ClustalW program (European Bioinformatics Institute, Cambridge, MA, USA). The residue numbering of *Yersinia* phytases begins with the initiating M of the signal sequence. Homology modeling of the *Yersinia* phytases was established using the Discovery studio 2.5.5 software (Accelrys, San Diego, CA, USA) with *E. coli* phytase (PDB: 1DKL) as the template. The relative solvent accessibility of the theoretical pepsin cleavage sites in the phytases was calculated using the I-TASSER program (http://zhanglab.ccmb.med.umich.edu/I-TASSER/). We selected three predicted pepsin cleavage sites, L99, L162, and E230, in YkAPPA and YeAPPA for mutagenesis at different locations and predicted solvent accessibility scores.

### Site-directed mutagenesis

Recombinant pEASY-T3 plasmids containing phytase genes *YkAPPA, YeAPPA*, and *YrAPPA* (GenBank accession no. EU203664, GU936684, and EF608455, respectively) were used to generate mutants^20^,^36^,^37^. A total of nineteen single, two double mutants, and two triple mutants were prepared by altering the side chain structures of the predicted pepsin cleavage sites (E230 to G, A, P, R, S, T, D, or K, and L162 to G, A, or V, and L99 to A) or introducing corresponding pepsin cleavage residues (V162 to L). Site-directed mutagenesis at specific positions was performed by overlap extension PCR with specific primers (Supplementary Table S1) as previously described^57^. The desired mutant genes were ligated into the pEASY-T3 vector (TransGen, Beijing, China) and confirmed by DNA sequencing.

### Phytase expression and purification

The gene fragments encoding the wild-type and mutant phytases without the signal peptide sequences were each digested with *Eco*RI and *Not*I and subsequently cloned into the expression vector pET-22b(+). His-tag fusion phytases were expressed in *E. coli* BL21(DE3) upon induction with 1 mM of isopropyl-β-D-thiogalactopyranoside (IPTG) at 24 °C. The crude phytase solutions were purified using a fast protein liquid chromatography system consisting of nickel-nitriloacetic (Ni-NTA) (Qiagen, Hilden, NRW, Germany) and diethylaminoethyl (DEAE) columns (GE Healthcare, Munich, Bavaria, Germany)^58^. The phytases were purified by 10% sodium dodecyl sulfate-polyacrylamide gel electrophoresis (SDS-PAGE) gels and stained with Coomassie brilliant blue R-250^59^. The total protein concentration was determined using the Bio-Rad protein assay kit (Hercules, CA, USA).

### Enzymatic activity assay

Specific activities of the wild-type and mutant phytases were measured using the ferrous sulfate-molybdenum blue method^38^. The reaction mixtures contained appropriately diluted enzymes, 1.5 mM of sodium phytate as the substrate, and 0.25 M sodium acetate (pH 4.5). After incubation at 37 °C for 30 min, the reactions were stopped by the addition of an equal volume of 10% (w/v) trichloroacetic acid and a double volume of the color reagent [1% (w/v) ammonium molybdate, 3.2% (v/v) sulfuric acid, and 7.2% (w/v) ferrous sulfate]. The amount of released inorganic phosphate was determined by the increase in the absorbance at 700 nm. One unit (U) of phytase activity was defined as the amount of enzyme required to liberate 1 μmol of phosphate per minute under the assay conditions. All reactions were performed in triplicate.

### Pepsin and trypsin digestion assays

Protease resistance was tested by incubating the wild-type and mutant phytases with or without pepsin (0.25 M glycine-HCl, pH 2.0) or trypsin (0.25 M Tris-HCl, pH 7.0) at 37 °C for 2 h at various protease/phytase mass ratios ranging from 1/1000 to 1/1. Aliquots were serially diluted for the residual activity assay as described above. The proteolytic reactions were stopped by the addition of 1.0 mM phenylmethylsulfonyl fluoride (PMSF). After denaturation by boiling in SDS-β-mercaptoethanol, the mixtures were run on an SDS-PAGE gels and stained with CBR-250^59^. The protein band intensity was estimated using the densitometric scanning software ImageJ (National Institutes of Health, Bethesda, MD, USA). The proteolytic rate (K_p_) of each enzyme was determined after 2 h of pepsin treatment by the decrease in the protein band intensity of the intact enzyme in an SDS-PAGE gel with increasing pepsin concentration, as described previously^31^. The half*-*life of enzyme proteolysis is the amount of pepsin needed to degrade half of the phytase after pepsin treatment for 2 h.

### Biochemical characterization of wild-type and mutant phytases

The pH profile of the wild-type and mutant phytases was determined at 37 °C for 30 min in various buffers: 0.25 M glycine-HCl (pH 1.0–3.5), sodium acetate-acetic acid (pH 3.5–6.0), Tris-HCl (pH 6.0–8.5), and glycine-NaOH (pH 8.5–12.0), respectively. The temperature optima were determined at each optimal pH by a 30-min incubation at 35 to 70 °C. The pH stability was evaluated after pre-incubation of the enzymes at 37 °C at pH 1.0–12.0 for 1 h or pH 1.0–4.0 for 2 h in the absence of substrate. The thermal stability was determined after pre-incubating the enzyme at the optimal pH and 60 °C without substrate for the desired interval by measuring the residual activity and the half-life of the enzyme for thermal incubation. The residual activities were measured as described above and indicated as a percentage of the highest value of phytase activity at the optimal pH and temperature. Untreated enzymes were considered as controls (100%).

The effect of metal ions and chemical reagents on the wild type and mutant enzymatic activity was evaluated by measuring the phytase activity in the presence of each reagent at a concentration of 1 mM.

### Kinetic measurements

The *K*_*m*_, *V*_*max*_, *k*_*cat*_, and *k*_*cat*_/*K*_*m*_ values were determined by a Lineweaver-Burk analysis after incubating each phytase with 0.0625–1.5 mM of sodium phytate in 0.25 M sodium acetate at the optimal pH at 37 °C for 10 min. The initial velocity was assayed by the hydrolysis of sodium phytate (0.125 mM) by phytase at 37 °C for 0–30 min.

### Enzymatic hydrolysis of corn meal

The hydrolysis ability of the wild-type and mutant phytases was evaluated using corn meal as the substrate under simulated gastric conditions^56^. The corn meal was treated at a body temperature of 37 °C and gastric pH 1.0–5.5 for 30 min as previously described^36^. The wild-type and mutant phytases (each at 1.0 U/g of corn meal) were each incubated in a 10% (w/v) corn meal solution with or without pepsin (0.25 M glycine-HCl, pH 2.0) at 37 °C for 1 h. The reaction was stopped by adding an equal volume of 10% (w/v) trichloroacetic acid, and the released inorganic phosphate was determined as previously described^60^.

## Additional Information

**How to cite this article:** Niu, C. *et al*. Engineering the residual side chains of HAP phytases to improve their pepsin resistance and catalytic efficiency. *Sci. Rep.* **7**, 42133; doi: 10.1038/srep42133 (2017).

**Publisher's note:** Springer Nature remains neutral with regard to jurisdictional claims in published maps and institutional affiliations.

## Supplementary Material

Supplementary Information

## Figures and Tables

**Table 1:** Proteolytic rate and half-life of wild type and mutant phytases after pepsin treatment^a^.

Enzymes	Proteolytic rate (10^−4^ g^−1^)	Half-life (10^−4^ g)
YkAPPA		
Wild-type	11.5 ± 0.2	0.007 ± 2E-4
E230G	0.0016 ± 2E-4	13.47 ± 1.91
E230P	0.0096 ± 2E-4	3.34 ± 0.21
E153R	0.026 ± 0.001	1.30 ± 0.05
E230A	0.0037 ± 2E-4	9.18 ± 0.14
E230S	0.101 ± 0.003	0.28 ± 0.03
E230D	1.46 ± 0.04	0.021 ± 0.001
E230T	1.47 ± 0.03	0.020 ± 8E-4
E230K	33.2 ± 2.81	0.002 ± 4E-4
L162G	0.024 ± 0.001	1.40 ± 0.04
L162A	0.050 ± 0.003	0.68 ± 0.02
L162V	0.11 ± 0.005	0.26 ± 0.008
L99A	0.79 ± 0.03	0.045 ± 0.01
L99A/L162G	0.009 ± 2E-4	3.54 ± 0.03
L99A/L162G/L230G	0.0007 ± 2E-4	25.21 ± 1.82
YeAPPA		
Wild-type	396 ± 14.2	0.0001 ± 2E-5
E230G	0.27 ± 0.008	0.17 ± 0.005
E230P	0.57 ± 0.002	0.052 ± 0.008
E230R	1.12 ± 0.03	0.033 ± 0.007
L162G	1.11 ± 0.03	0.031 ± 0.005
L162A	2.88 ± 0.08	0.012 ± 0.002
L162V	4.50 ± 0.13	0.009 ± 2E-4
L99A	7.24 ± 0.14	0.004 ± 2E-4
L99A/L162G	0.71 ± 0.05	0.069 ± 0.02
L99A/L162G/L230G	0.11 ± 0.002	0.44 ± 0.07

^a^Proteolysis rate and half-life of each phytase were determined after pepsin treatment at 37 °C for 2 h as a function of pepsin concentrations.

**Table 2:** Effect of pH and temperature on the activity and stability of wild type and mutant phytases.

Enzyme	pH optimum	Temperature optimum (°C)	pH stability	Thermostability	
			Residual activity (%)^a^ at pH 1.0 for 1 h	Residual activity (%)^a^ at 60 °C for 30 min	Half-life (min) at 60 °C
YkAPPA					
WT	4.5	55	pH 1.0–1.5, 63.7–77.0; pH 2.0–10.0, >90.5	16.1 ± 0.41	12.5 ± 0.52
E230G	4.5	55	pH 1.0–1.5, >92.4; pH 2.0–10.0, >99.3	34.7 ± 1.23	21.7 ± 0.82
E230A	4.5	55	pH 1.0–1.5, >87.0; pH 2.0–10.0, >99.2	16.0 ± 0.73	12.4 ± 0.33
E153P	4.5	60	pH 1.0–1.5, <78.0; pH 2.0–10.0, >87.8	41.7 ± 1.74	25.6 ± 0.48
E230R	4.0	55	pH 1.0–1.5, >86.8; pH 2.0–10.0, >94.6	22.6 ± 0.83	14.5 ± 0.37
E230S	4.5	55	pH 1.0–1.5, <78.7; pH 2.0–10.0, >89.2	33.6 ± 1.82	19.9 ± 0.77
E230T	4.5	55	pH 1.0–1.5, <78.3; pH 2.0–10.0, >91.1	33.4 ± 2.42	19.5 ± 0.73
E230D	4.5	55	pH 1.0–1.5, <76.5; pH 2.0–10.0, >90.8	16.5 ± 0.76	12.7 ± 0.38
E230K	4.5	55	pH 1.0–1.5, >80.0; pH 2.0–10.0, >94.6	16.1 ± 0.63	12.5 ± 0.52
L162V	4.5	60	pH 1.0–1.5, >83.5; pH 2.0–10.0 >93.6	30.5 ± 1.45	17.9 ± 0.39
L162A	4.5	55	pH 1.0–1.5, >85.1; pH 2.0–10.0 >93.2	16.8 ± 0.43	12.8 ± 0.44
L162G	4.5	55	pH 1.0–1.5, >89.9; pH 2.0–10.0 >99.7	16.6 ± 0.51	12.7 ± 0.37
L99A	4.5	55	pH 1.0–1.5, <78.1; pH 2.0–10.0 >92.1	15.8 ± 0.48	12.1 ± 0.58
L99A/L162G	4.5	55	pH 1.0–1.5, >90.3; pH 2.0–10.0 >99.2	16.2 ± 0.55	12.6 ± 0.62
L99A/L162G/E230G	4.5	55	pH 1.0–1.5, >90.3; pH 2.0–10.0 >99.2	35.1 ± 1.31	21.9 ± 0.77
YeAPPA					
WT	5.0	45	pH 1.0–2.0, <12.3; pH 3.0–9.0, >88.7	0.6 ± 0.02	1.1 ± 0.04
E230G	5.0	45	pH 1.0–2.0, >32.1; pH 3.0–9.0, >99.6	21.4 ± 0.94	13.8 ± 0.43
E230P	5.0	50	pH 1.0–2.0, >24.3; pH 3.0–9.0, >99.4	11.5 ± 0.82	12.9 ± 0.46
E230R	4.0	45	pH 1.0–2.0, >29.7; pH 3.0–9.0, >99.3	8.9 ± 0.04	11.3 ± 0.37
L162V	5.0	45	pH 1.0–2.0, >13.5; pH 3.0–9.0, >92.3	9.5 ± 0.04	11.7 ± 0.42
L162A	5.0	45	pH 1.0–2.0, >17.6; pH 3.0–9.0, >96.4	0.6 ± 0.04	1.1 ± 0.24
L162G	5.0	45	pH 1.0–2.0, >20.1; pH 3.0–9.0, >99.4	0.6 ± 0.004	1.1 ± 0.31
L99A	5.0	45	pH 1.0–2.0, >13.3; pH 3.0–9.0, >90.1	0.7 ± 0.004	1.1 ± 0.29
L99A/L162G	5.0	45	pH 1.0–2.0, >21.8; pH 3.0–9.0, >99.6	0.6 ± 0.005	1.1 ± 0.27
L99A/L162G/E230G	5.0	45	pH 1.0–2.0, >38.8; pH 3.0–9.0, >99.2	21.7 ± 0.89	13.9 ± 0.61

^a^The phytase activity towards sodium phytate (1.5 mM) at 37 °C for 30 min was regarded as 100%. The residual activity was indicated as percentage of activity of untreated enzyme.

**Table 3:** Kinetics of wild type and mutant phytases^a^.

Enzyme	*K*_m_ (mM)	*V*_max_ (U mg^−1^)	*k*_cat_ (S^−1^)	*k*_cat_/*K*_m_ (S^−1^ mM^−1^)
YkAPPA				
Wild-type	0.09 ± 0.01	3554 ± 43	2719 ± 33	29423 ± 299
E230G	0.10 ± 0.03	7097 ± 46	5429 ± 125	61690 ± 336
E230A	0.09 ± 0.02	4533 ± 37	3468 ± 139	37685 ± 227
E230P	0.08 ± 0.04	3177 ± 61	2430 ± 133	29833 ± 192
E230R	0.11 ± 0.03	4329 ± 48	3312 ± 147	29883 ± 318
E230S	0.09 ± 0.01	3795 ± 32	2903 ± 98	29298 ± 137
E230T	0.08 ± 0.02	3247 ± 28	2484 ± 69	29767 ± 125
E230D	0.09 ± 0.02	3587 ± 42	2744 ± 122	29088 ± 204
E230K	0.09 ± 0.01	3564 ± 37	2726 ± 43	30118 ± 164
L162V	0.10 ± 0.05	3646 ± 5	2789 ± 117	29310 ± 522
L162A	0.10 ± 0.02	3917 ± 23	2996 ± 102	29537 ± 154
L162G	0.09 ± 0.01	6321 ± 36	4836 ± 132	46084 ± 238
L99A	0.10 ± 0.02	3906 ± 52	2988 ± 105	29651 ± 283
L99A/L162G	0.10 ± 10.4	3920 ± 41	2999 ± 33	29198 ± 266
L99A/L162G/E230	0.09 ± 0.04	3587 ± 49	2703 ± 32	29652 ± 186
YeAPPA				
Wild-type	0.19 ± 0.01	6.4 ± 0.01	4.9 ± 0.13	26.1 ± 0.44
E230G	0.19 ± 0.01	15.6 ± 0.03	12.0 ± 0.25	63.8 ± 1.3
E230P	0.19 ± 0.03	6.7 ± 0.19	5.1 ± 0.13	26.4 ± 1.5
E230R	0.18 ± 0.02	6.3 ± 0.04	4.8 ± 0.35	26.0 ± 1.8
L162V	0.19 ± 0.01	9.5 ± 0.01	7.3 ± 0.19	38.4 ± 1.1
L162A	0.19 ± 0.04	6.5 ± 0.12	5.0 ± 0.75	26.6 ± 3.8
L162G	0.19 ± 0.05	10.7 ± 0.08	8.2 ± 0.66	42.7 ± 5.7
L99A	0.20 ± 0.04	6.9 ± 0.05	5.3 ± 0.35	26.3 ± 2.6
L99A/L162G	0.18 ± 0.04	6.0 ± 0.02	4.6 ± 0.45	26.5 ± 2.1
L99A/L162G/E230	0.19 ± 0.01	6.5 ± 0.13	5.0 ± 0.1	25.7 ± 1.9

^a^Enzyme kinetic assays were performed on substrate sodium phytate in 0.25 M sodium acetate buffer at the optimal pH and 37 °C for 10 min in the presence of wild type and mutants. Each value presents the mean ± SD (n = 3) from three independent tests.
