# Corrigendum: Engineering the residual side chains of HAP phytases to improve their pepsin resistance and catalytic efficiency

**DOI:** 10.1038/srep46871

**Published:** 2017-06-30

**Authors:** Canfang Niu, Peilong Yang, Huiying Luo, Huoqing Huang, Yaru Wang, Bin Yao

Scientific Reports 7: Article number: 4213310.1038/srep42133; published online: 02 10 2017; updated: 06 30 2017

In the original version of this Article, there were errors in the affiliation which was incorrectly listed as ‘Key Laboratory for Feed Biotechnology of the Ministry of Agriculture, Feed Research Institute, Chinese Academy of Agricultural Sciences, Beijing 100081, People’s Republic of China’. The correct affiliation is listed below:

National Engineering Research Center of Biological Feed, Key Laboratory for Feed Biotechnology of the Ministry of Agriculture, Feed Research Institute, Chinese Academy of Agricultural Sciences, Beijing 100081, People’s Republic of China.

These errors have now been corrected in the PDF and HTML versions of the Article.

