# Supplementary material for: Engineering the residual side chains of HAP phytases to improve their pepsin resistance and catalytic efficiency
Source: Sci Rep. 2017 Feb 10;7:42133. doi: 10.1038/srep42133 (PMC5301473; doi:10.1038/srep42133)
Supplement: Supplementary Information [file srep42133-s1.pdf]

RESEARCH ARTICLE

Engineering the residual side chains of HAP phytases to improve their pepsin resistance and catalytic efficiency

Canfang Niu[1], Peilong Yang, Huiying Luo, Huoqing Huang, Yaru Wang, Bin Yao[1]

Key Laboratory for Feed Biotechnology of the Ministry of Agriculture, Feed Research Institute, Chinese Academy of Agricultural Sciences, Beijing 100081, People's Republic of China

[1]Corresponding authors. Address: Key Laboratory for Feed Biotechnology of the Ministry of Agriculture, Feed Research Institute, Chinese Academy of Agricultural Sciences, No. 12 Zhongguancun South Street, Beijing 100081, P. R. China. Tel.: +86 10 62169913; fax: +86 10 82106054.

E-mail addresses: niucanfang@163.com (CN); binyao@caas.cn (BY).

Table S1 The protease resistance of phytases and the corresponding residues

Phytase origin	Residues at corresponding positions	Residual activity (%) [a]		References
		Pepsin	Trypsin	
Yersinia enterocolitica	L99, L162, E230	3.1 at the ratio of 1/1000	54.1 at the ratio of 1/20	This study
Y. kristensenii	L99, L162, E230	21.2 at the ratio of 1/100	99.9 at the ratio of 1/20	This study
Y. rohdei	L99, V230, D230	70.2 at the ratio of 1/1	104.9 at the ratio of 1/1	Huang et al., 2008; Niu et al., 2015
Aspergillus ficuum	L82, T146, S211	98.9 at the ratio of 1/1	97.1 at the ratio of 1/1	Zhang et al., 2010
A. japonicus	I102, V166 ,G248	100 at the ratio of 1/50	20 at the ratio of 1/200	Promdonkoy et al., 2009

[a] Residual activity was indicated as percentage of activity of untreated phytases after incubation with pepsin at pH 2.0 or trypsin at pH 7.0 and 37°C for 2 h at various protease/phytase mass ratios (Huang, H. *et al*. *Microbiol*. *Biotechnol*. **80,** 417–426, 2008; Niu, C. *et al*. *Appl*. *Environ*. *Microbiol*. **82,** 1004–14, 2015; Zhang, G. *et al*. *Bioresour*. *Technol*. **101,** 4125–31, 2010; Promdonkoy, P. *et al. FEMS. Microbiol. Lett.* **290,** 18–24, 2009).

Table S2 The solvent accessibility of the mutated sites in phytases

	YkAPPA			YeAPPA			YrAPPA		
Mutated sites	99	162	230	99	162	230	99	162	230
Solvent exposed residues	L	L	E	L	L	E	L	V	D
Predicted solvent accessibility	1	2	4	1	2	4	1	2	4

Table S3 Primers for YkAPPA, YeAPPA and YrAPPA and their mutants

Enzymes	Mutation	Primer sequences[a]
YkAPPA	Wild-type[b]	Forward: 5’-cgc*gaattc*gcaccgcttgcagcacaatctac-3’/Reverse: 5’-gat*gcggccgc*ttaaatatggcaggctggctcg-3’
	E230G	Forward: 5’-tcgaggtaaataaaggcgggacaaaagtctc-3’/Reverse: 5’-gagacttttgtcccgcctttatttacctcga-3’
	E230A	Forward: 5’-tcgaggtaaataaagcggggacaaaagtctc-3’/Reverse: 5’-gagacttttgtccccgctttatttacctcga-3’
	E230P	Forward: 5’-tcgaggtaaataaaccggggacaaaagtctc-3’/Reverse: 5’-gagacttttgtccccggtttatttacctcga-3’
	E230R	Forward: 5’-tcgaggtaaataaacgtgggacaaaagtctc-3’/Forward: 5’-gagacttttgtcccacgtttatttacctcga-3’
	E230S	Forward: 5’-tcgaggtaaataaatctgggacaaaagtctc-3’/Reverse: 5’-gagacttttgtcccagattatttacctcga-3’
	E230T	Forward: 5’-tcgaggtaaataaaaccgggacaaaagtctc-3’/Reverse: 5’-gagacttttgtcccggttttatttacctcga-3’
	E230D	Forward: 5’-tcgaggtaaataaagatgggacaaaagtctc-3’/Reverse: 5’-gagacttttgtcccatctttatttacctcga-3’
	E230K	Forward: 5’-tcgaggtaaataaaaaggggacaaaagtctc-3’/Reverse: 5’-gagacttttgtccccttttatttacctcga-3’
	L162G	Forward: 5’-cgggggtatgtaaaggcgacccagagaaaac-3’/Reverse: 5’-gttttctctgggtcgcctttacatacccccg-3’
	L162A	Forward: 5’-cgggggtatgtaaagcggacccagagaaaac-3’/Reverse: 5’-gttttctctgggtccgctttacatacccccg-3’
	L162V	Forward: 5’-cgggggtatgtaaagtggacccagagaaaac-3’/Reverse: 5’-gttttctctgggtccactttacatacccccg-3’
	L99A	Forward: 5’-tccgcagctatggggcgttaccggcggggtg-3’/Reverse: 5’-caccccgccggtaacgccccatagctgcgga-3’
YeAPPA	Wild-type[b]	Forward: 5’-cgc*gaattc*gccccgattgctacaccgcc-3’/Reverse: 5’-gat*gcggccgc*ttaaatatggcaggctggctcga-3’
	E230G	Forward: 5’-ttaaggtaaacgaaggcggtactaaagtttc-3’/Reverse: 5’-gaaactttagtaccgccttcgtttaccttaa-3’
	E230P	Forward: 5’-ttaaggtaaacgaaccgggtactaaagtttc-3’/Reverse: 5’-gaaactttagtacccggttcgtttaccttaa-3’
	E230R	Forward: 5’-ttaaggtaaacgaacgtggtactaaagtttc-3’/Reverse: 5’-gaaactttagtaccacgttcgtttaccttaa-3’
	L162G	Forward: 5’-cgggggtctgtaaaggcgactcagcgaaaac-3’/Reverse: 5’-gttttcgctgagtcgcctttacagacccccg-3’
	L162A	Forward: 5’-cgggggtctgtaaagcggactcagcgaaaac-3’/Reverse: 5’-gttttcgctgagtccgctttacagacccccg-3’
	L162V	Forward: 5’-cgggggtctgtaaagtggactcagcgaaaac-3’/Reverse: 5’-gttttcgctgagtccactttacagacccccg-3’
	L99A	Forward: 5’-tccgcagccaaggggcgttgtctgcggggtg-3’/Reverse: 5’-caccccgcagacaacgccccttggctgcgga-3’
YrAPPA	Wild-type[b]	Forward: 5’-ctt*gaattc*gccccggtgataaccgcacc-3’/Reverse: 5’-ta*gcggccgc*ttaaatatggcaggctggctc-3’
	V162L	Forward: 5’-gtgtttgtaagctagatttagcccaaac-3’/Reverse: 5’-gtttgggctaaatctagcttacaaacac-3’

[a] All mutation sites in primers are underlined and the restriction enzyme sites in the primes are italic.

[b] These genes and enzymes are the products of our previous studies (Fu, D. *et al*. *Appl*. *Microbiol*. *Biotechnol*. **90,** 1295–1302, 2011; Fu, D. *et al*. *Enzyme*. *Microb*. *Tech*. **42,** 499–505, 2008; Huang, H. *et al*. *Microbiol*. *Biotechnol*. **80,** 417–426, 2008).

Table S4 Changes in pepsin resistance of phytases by the pepsin cleavage site substitutions (fold) [a]

Enzymes		Mass ratio of pepsin/phytase							
		1/1000	1/500	1/200	1/100	1/40	1/20	1/10	1/1
YkAPPA	E230G	1.5 ± 0.01	2.0 ± 0.02	3.4 ± 0.05	5.5 ± 0.06	15.3 ± 0.29	74.0 ± 2.4	327 ± 7.5	546 ± 9.4
	E230A	1.4 ± 0.03	1.9 ± 0.03	3.3 ± 0.04	5.1 ± 0.04	14.1 ± 0.39	67.3 ± 2.2	273 ± 8.9	482 ± 7.6
	E230P	1.5 ± 0.02	1.9 ± 0.04	3.1 ± 0.01	4.7 ± 0.05	11.9 ± 0.31	56.7 ± 2.5	216 ± 5.8	369 ± 8.2
	E230R	1.3 ± 0.02	1.7 ± 0.03	2.7 ± 0.02	3.7 ± 0.08	9.5 ± 0.27	43.2 ± 1.9	155 ± 3.5	237 ± 5.3
	E230S	1.3 ± 0.01	1.5 ± 0.03	2.3 ± 0.06	3.0 ± 0.11	7.0 ± 0.18	30.0 ± 1.6	105 ± 2.8	161 ± 4.3
	E230T	1.1 ± 0.03	1.3 ± 0.02	1.6 ± 0.04	1.7 ± 0.03	3.7 ± 0.13	11.1 ± 0.43	16.5 ± 0.81	15.4 ± 0.63
	E230D	1.1 ± 0.02	1.3 ± 0.02	1.7 ± 0.02	1.8 ± 0.02	3.6 ± 0.08	10.4 ± 0.34	16.1 ± 0.74	14.7 ± 0.43
	E230K	-1.2 ± 0.01	-1.4 ± 0.04	-1.5 ± 0.03	-2.5 ± 0.01	-2.8 ± 0.01	--	--	--
	L162V	1.2 ± 0.03	1.5 ± 0.02	2.2 ± 0.03	2.7 ± 0.05	6.9 ± 0.13	29.7 ± 1.4	ND	ND
	L162A	1.3 ± 0.02	1.7 ± 0.05	2.5 ± 0.04	3.1 ± 0.04	8.6 ± 0.25	37.5 ± 2.1	ND	ND
	L162G	1.4 ± 0.01	1.8 ± 0.02	2.7 ± 0.02	3.5 ± 0.08	9.9 ± 0.31	44.1 ± 1.6	ND	ND
	L99A	1.1 ± 0.02	1.3 ± 0.01	1.6 ± 0.04	1.9 ± 0.03	4.1 ± 0.16	14.3 ± 0.41	ND	ND
	L99A/L162G	1.4 ± 0.03	1.9 ± 0.02	3.0 ± 0.03	4.4 ± 0.09	12.3 ± 0.34	60.0 ± 1.8	ND	ND
	L99A/L162G/E230G	1.5 ± 0.02	2.0 ± 0.03	3.5 ± 0.02	5.6 ± 0.12	15.7 ± 0.42	81.6 ± 2.7	360 ± 4.6	664 ± 9.8
YeAPPA	E230G	82.4 ± 2.4	237 ± 6.7	284 ± 3.7	318 ± 5.9	447 ± 7.8	--	--	--
	E230P	70.0 ± 2.6	205 ± 6.2	234 ± 3.5	265 ± 5.3	315 ± 6.7	--	--	--
	E230R	65.6 ± 2.3	168 ± 3.2	193 ± 3.3	203 ± 3.7	265 ± 5.5	--	--	--
	L162V	47.3 ± 1.9	118 ± 2.9	109 ± 2.2	110 ± 2.8	109 ± 2.9	--	ND	ND
	L162A	60.0 ± 2.1	140 ± 3.3	149 ± 2.9	154 ± 3.1	169 ± 3.4	--	ND	ND
	L162G	62.1 ± 2.2	172 ± 6.4	196 ± 4.2	211 ± 3.9	292 ± 5.7	--	ND	ND
	L99A	24.8 ± 1.3	62.8 ± 1.9	57.6 ± 4.1	60.4 ± 1.8	75.9 ± 2.6	--	ND	ND
	L99A/L162G	73.4 ± 2.3	209 ± 4.7	242 ± 2.1	270 ± 5.2	355 ± 6.3	--	ND	ND
	L99A/L162G/E230G	103 ± 2.8	311 ± 5.9	410 ± 8.4	456 ± 8.6	724 ± 13	--	ND	ND
YrAPPA	V162L	-1.2 ± 0.03	-1.3 ± 0.02	-1.5 ± 0.03	-1.6 ± 0.01	-1.7 ± 0.02	-2.3 ± 0.01	ND	ND

[a] “fold” indicates the ratio of the residual activity of the pepsin cleavage site substitution mutant to that of the wild type after pepsin treatment at pH 2.0 and 37°C for 2 h at various pepsin/phytase mass ratios. Each value represents the mean ± SD (n=3) from three independent experiments. “-” indicates a decrease in pepsin resistance. “--” indicates that the wild-type phytase lost all activity after pepsin treatment for 2 h. “ND”, not detected.

Table S5 Effect of various chemicals on the activity of wild-type and mutant phytases

Chemicals (1 mM)	Phytase activity (%)											
	YkAPPA						YeAPPA					
	Wild-type	L99A	E230G	E230A	L162G	L162V	Wild-type	L99A	E203G	E230A	L162A	L162V
CK	100 ± 2	100 ± 1	100 ± 2	100 ± 1	100 ± 1	100 ± 2	100 ± 1	100 ± 2	100 ± 2	100 ± 2	100 ± 1	100 ± 1
Na^+	100 ± 2	99 ± 2	103 ± 1	101 ± 1	100 ± 1	98 ± 1	103 ± 1	99 ± 1	100 ± 1	101 ± 2	101 ± 1	101 ± 2
K^+	98 ± 3	97 ± 1	95 ± 1	97 ± 2	99 ± 2	97 ± 2	99 ± 2	98 ± 1	100 ± 1	100 ± 1	98 ± 2	99 ± 2
Ca^{2+}	94 ± 2	95 ± 1	95 ± 1	99 ± 1	95 ± 1	96 ± 2	130 ± 2	126 ± 3	124 ± 2	123 ± 2	120 ± 1	126 ± 1
Co^{2+}	101 ± 2	102 ± 2	100 ± 1	99 ± 2	99 ± 1	100 ± 1	106 ± 2	107 ± 1	93 ± 2	105 ± 1	103 ± 1	104 ± 1
Li^+	98 ± 3	100 ± 2	99 ± 1	98 ± 1	99 ± 2	97 ± 2	103 ± 1	105 ± 3	104 ± 1	104 ± 2	99 ± 1	102 ± 2
Hg^{2+}	9 ± 1	10 ± 1	9 ± 2	11 ± 2	42 ± 1	39 ± 1	9 ± 1	10 ± 1	11 ± 0	10 ± 1	54 ± 1	48 ± 1
Fe^{3+}	7 ± 3	9 ± 2	8 ± 1	7 ± 2	29 ± 1	27 ± 1	78 ± 1	75 ± 3	79 ± 1	80 ± 2	95 ± 2	91 ± 1
Cr^{3+}	91 ± 1	92 ± 3	90 ± 1	92 ± 1	92 ± 2	93 ± 1	99 ± 2	97 ± 3	99 ± 1	98 ± 1	100 ± 2	98 ± 1
Ni^{2+}	101 ± 2	98 ± 3	100 ± 2	100 ± 2	100 ± 1	100 ± 1	101 ± 2	99 ± 1	99 ± 2	100 ± 1	101 ± 1	100 ± 1
Cu^{2+}	88 ± 3	89 ± 2	88 ± 1	90 ± 2	99 ± 1	97 ± 1	6 ± 2	5 ± 2	7 ± 1	8 ± 1	47 ± 1	45 ± 2
Mg^{2+}	96 ± 3	95 ± 1	95 ± 2	94 ± 1	96 ± 1	94 ± 1	97 ± 1	99 ± 1	97 ± 1	94 ± 1	99 ± 2	96 ± 1
Mn^{2+}	91 ± 2	92 ± 3	90 ± 1	90 ± 1	93 ± 1	93 ± 2	104 ± 2	104 ± 1	100 ± 2	101 ± 2	100 ± 3	100 ± 2
Zn^{2+}	48 ± 2	49 ± 2	87 ± 2	89 ± 1	69 ± 2	67 ± 2	37 ± 1	39 ± 2	80 ± 1	68 ± 2	54 ± 2	50 ± 1
Pd^{2+}	25 ± 2	26 ± 2	26 ± 1	25 ± 1	27 ± 1	28 ± 1	78 ± 1	77 ± 3	80 ± 2	81 ± 1	79 ± 1	82 ± 1
Ag^+	74 ± 3	76 ± 3	78 ± 2	75 ± 1	90 ± 2	86 ± 2	62 ± 1	64 ± 3	61 ± 1	60 ± 2	84 ± 1	78 ± 1
SDS	0 ± 1	0 ± 2	0 ± 1	0 ± 1	0 ± 1	0 ± 1	0 ± 2	0 ± 1	0 ± 1	0 ± 2	0 ± 1	0 ± 1
EDTA	99 ± 2	96 ± 3	97 ± 2	98 ± 2	100 ± 1	99 ± 2	103 ± 2	99 ± 2	99 ± 2	99 ± 1	100 ± 1	101 ± 1

[a] The reactions were carried out in triplicate in 0.25 M sodium acetate buffer, optimal pH, with 1.5 mM sodium phytate as the substrate at 37°C. Data present means ± SDs relative to the control treatment without chemical addition.

Fig. S1 Sequence alignment of the amino acids possibly responsible for pepsin resistance of five phytases. Identical residues are shaded in black. The key residues are L99 (▲), L162 (●), and E230 (■). Microbial source (GenBank accession number): YeAPPA, *Yersinia enterocolitica* (GU936684); YkAPPA, *Y. kristensenii* (EU203664); YrAPPA, *Y. rohdei* (EF608455); AfphyB, *Aspergills ficuum* (GQ304756); AjphyA, *A. japonicus* (EU786166).

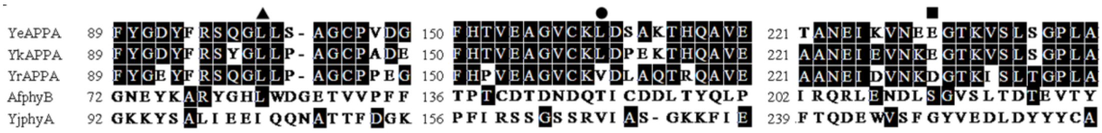

Fig. S2 SDS-PAGE analysis of the wild-type and mutant *Yersinia* phytases produced in *E. coli*

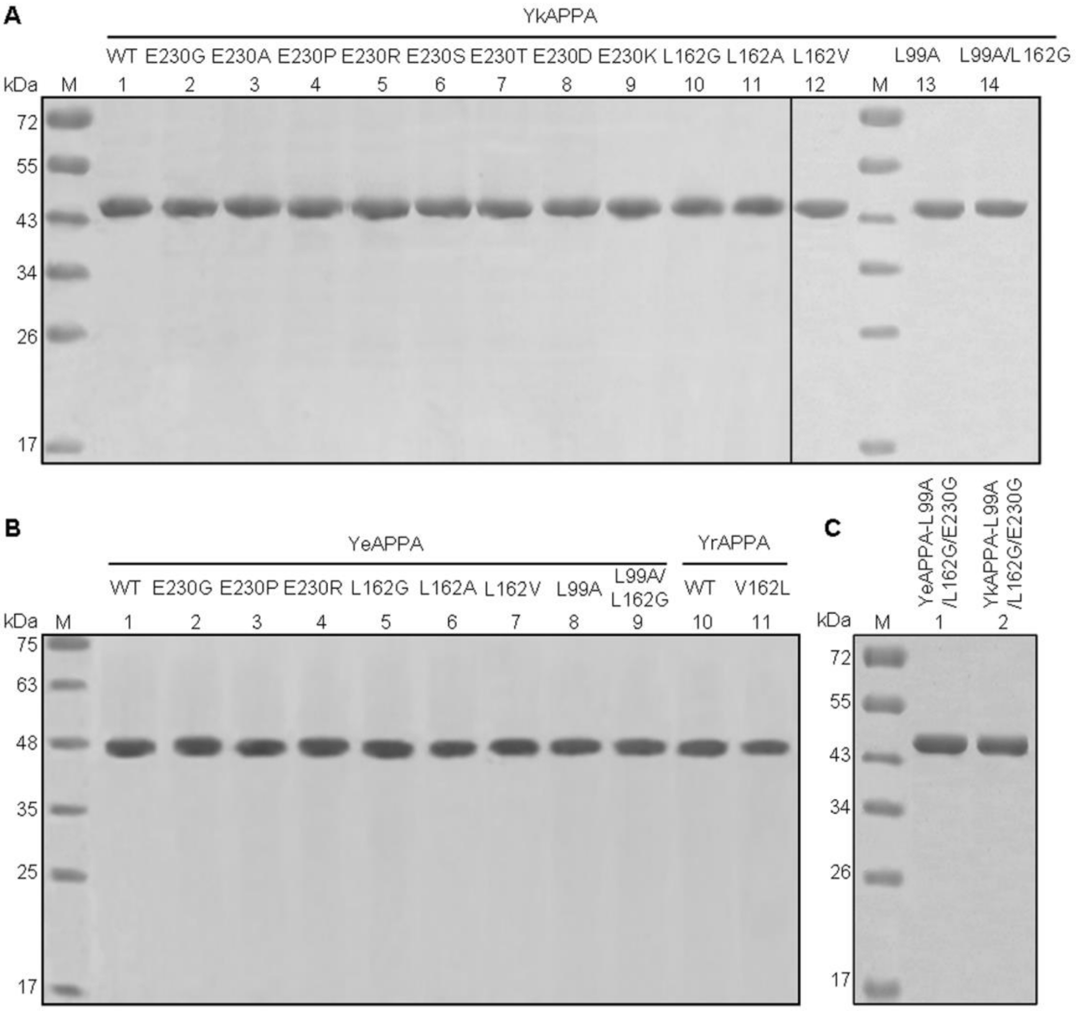

(A) Lanes: M, the protein markers; 1 to 14, wild-type YkAPPA and its mutants E230G, E230A, E230P, E230R, E230S, E230T, E230D, E230K, L162G, L162A, L162V, L99A, L99A/L162G. (B) Lanes: 1 to 9, wild-type YeAPPA and its mutants E230G, E230P, E230R, L162G, L162A, L162V, L99A, and L99A /L162G; 10 and 11, wild-type YrAPPA and its mutant V162L. (C) Lanes 1 and 2, L99A/L162G/E230G mutants of both phytases.
